# Supplementary material for: Dusky-like Is Critical for Morphogenesis of the Cellular Protuberances and Formation of the Cuticle in Henosepilachna vigintioctopunctata
Source: Biology (Basel). 2023 Jun 15;12(6):866. doi: 10.3390/biology12060866 (PMC10295638; doi:10.3390/biology12060866)
Supplement: Supplementary file 1 [file biology-12-00866-s001.zip › biology-2401330-supplementary.pdf]

Supplementary data

Dusky-like Is Critical for Morphogenesis of the Cellular Protuberances and Formation of the Cuticle in *Henosepilachna vigintioctopunctata*

Yuxing Zhang, Qiao Tan, Mengjiao Lin, Chenhui Shen, Lin Jin and Guoqing Li *

Education Ministry Key Laboratory of Integrated Management of Crop Diseases and Pests, State & Local Joint Engineering Research Center of Green Pesticide Invention and Application, Department of Entomology, College of Plant Protection, Nanjing Agricultural University, Nanjing 210095, China; 2020202042@stu.njau.edu.cn (Y.Z.); 2020102083@stu.njau.edu.cn (Q.T.); 2020102082@stu.njau.edu.cn (M.L.); 2019202031@njau.edu.cn (C.S.); jinlin@njau.edu.cn (L.J.)

* Correspondence: ligq@njau.edu.cn; Tel./Fax: +86-25-84395248

Table S1. A list of primers used for RT-PCR, dsRNA synthesis and qRT-PCR

Fragment name	Forward primer (5’-3’)	Reverse primer (5’-3’)
**RT-PCR**		
HvDusky-like	ATACTGGTACAGCAAACAAA	CTCTGCATTGTCGTAACCT
**dsRNA synthesis**		
ds*HvDusky-like*-F1R1	TAATACGACTCACTATAGGGATACAAGTGGGCAAAGG	TAATACGACTCACTATAGGGACTACGGAAGCAGATGG
ds*HvDusky-like*-F2R2	TAATACGACTCACTATAGGGGCCCCTTGCTACACCAGATA	TAATACGACTCACTATAGGGCCACCATAACTAGCGGCATT
ds*egfp*	TAATACGACTCACTATAGGGAAGTTCAGCGTGTCCG	TAATACGACTCACTATAGGGCACCTTGATGCCGTTC
**qRT-PCR**		
q*HvDusky-like*	TTCAGCAGCAGATCCAGAGA	CCTCTAGCATGGACGAAAGC
q*HvRPS18*	CGCAATCAAAGGTGTTGGAAG	GCCTAGGGTTGGCCATAATAG
q*HvRPL13*	AGCATCCTTCGCTCGTTTAG	TTCGACAACCTGCCATTAGG

HvDyl

ATGACAAGGTCACTTCATATGTTGAGCATCGCTATACTTGCAGTGTTATTTTTAAGGAACACACGC
TGTGATGCACCAATACCAGACTCTGCGTCTCTTCCATTAGAACACAGAGGAATATACGGACCACCA
CTTTCAGGTCCTGCTTATGCTCCTCCGTCCGCTCTCATACAGAGCGGAGGTGGTGGTGGAGGTTCA
GATGATCCATGGCCCCTTGCTACACCAGATATGCCCCAAATCAAACATTTGCAAGTACAGTGTGAA
AAAACACATATGAGGGTGAATATTGAATTCGACCGCCCTTTCTATGGTATGATATTCTCCAAGGGT
TTCTACAGTGATCACCACTGTGTACATTTGAAACCTGGTACTGGTCACTTGAGCGCCACATTTGAA
ATATTCTTGAACAGTTGCGGCATGTCTTCATCGGCCAATCATAATGCCGCTAGTTATGGTGGACCA
ACACCTAGTGGAAGCTATGTAGAGAACACAATTATAATTCAGTACGATCCATTCGTACAAGAAGTT
TGGGATCAAGCAAGAAAACTACGTTGTACCTGGTATGATTTCTATGAGAAGGCTGTTACTTTCAGA
CCATTCCAAGTAGATATGCTACATGCAGTTACTGCTAACTTCTTGGGCGATAATTTACAATGCTGG
ATGCAAATACAAGTGGGCAAAGGACCCTGGGCTTCTGAAGTATCTGGAATAGTGAAAATTGGACAA
ACAATGACAATGGTACTCGCTATAAAAGATGATGAAAATAAGTTCGATATGTTGGTCAGAAACTGT
GTGGCTCATGATGGAAAGAGAGCACCAATTCAATTGGTTGATCAGTATGGTTGCGTTGTTAGACCT
AAAATTATGAGTAAATTCCAGAAGATAAAGAACTTCGGACCATCTGCTTCCGTAGTGTCATTCGCT
TATTTCCAAGCATTCAAATTCCCAGATTCAATGAATGTTCATTTCCAATGTGTAATTCAAGTCTGC
AGATACAATTGTCCAGAACCTAAATGCGGGCATGGTCTTGGATTGTCGAATGAGTATGGCGTTCCT
GCATTAGGTGGCAGCGGACATGGAGGTAATTTGCATGCTGAATATGGACCTCCACCACTTCCAGAA
TACAACGGAGTTTCACCTGCATTCCCAGACCCCAGACACCCTTCAGGCCCAACTGGAGCTTATTCT
GAACCGAATCCTGATGTTGTTCCTGCTCCACAAGCTCAAACATCTTCCTCATCGACTTCGAATCCG
TCAAATCTTTCTTCTCTAGCACCGCAAAGTAACTCTCAGACAGGAAACGATTTGCATTTACCTCCA
CCACCTCCACCTGGACACCACGGTCAATACAACACTGTGAAGAGGAAGAGCGATATCGAAGGAGAA
TTCGAAAACAACTTAGCTACCTTAGGTGGACGTCCACGGACTTTGGAAAATCCTGCACAACTCCAG
GGTGTGAGACGTAGGCGCAGTCCTGACACATTCAACATTGTGGTTAGTCACGTCTATAAAAGAGAA
GCTCAAGAAATGACTGACGTTAATACTAGTAGGATCATTCAAGTGGTTGCACCTGGAGATGTTAAC
TTTGCTCTAAATTCTGCTCAAAACAACGAAACCGTAGTTATTCAATCAGCTTCAGCAGCAGATCCA
GAGACAATTTGTATGTCCATCCCTAGTTTTGTCGCTGGACTTGTCATGCTCCTTTTAGTGCTTATC
GTGTCGAGTTTAGTGGCTGCCTTCTTGTTCGTGAGAGTACGCGCCATTGATAGGAAAGGAGCAGCA
CCAGCTTTCGTCCATGCTAGAGGTTACGACAATGCAGAGTTCGTCAAAGTACCAAATTAG

Figure S1. The positions of the primary and secondary dsRNA on the mRNA. The green represents the position of primary dsRNA (ds*HvDusky-like*-F1R1), The yellow represents the position of secondary dsRNA (ds*HvDusky-like*-F2R2).

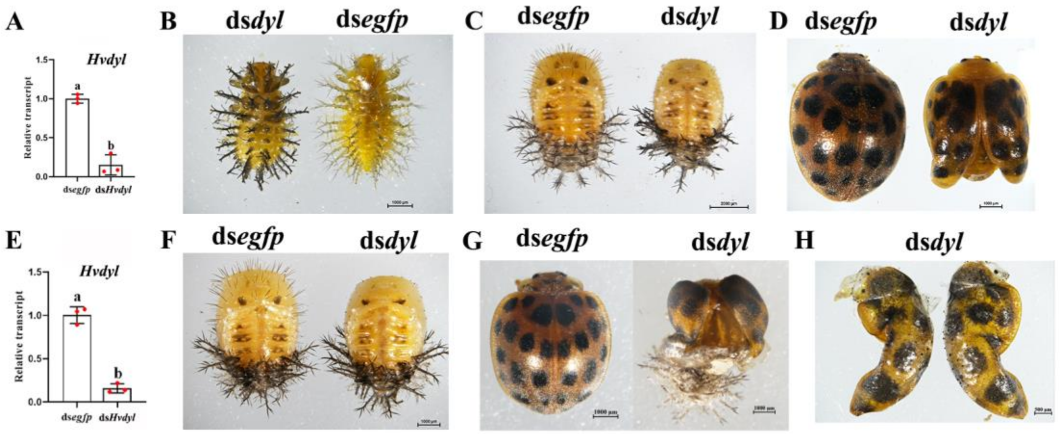

Figure S2. Knockdown of *Hvdyl* with secondary dsRNA at the third- and fourth-instar larval stage impairs growth of the cellular protuberances and the formation of cuticle in *Henosepilachna vigintioctopunctata*. The newly molted third- and fourth-instar larvae were injected with 300 ng ds*egfp* or ds*dyl* and then transferred to potato foliage. Two days after third-instar larvae injection, transcript levels of *Hvdyl* were measured (A). The phenotypes of fourth-instar larvae (B), pupae (C) and adults (D) were observed and imaged (third-instar larvae injection). Two days after fourth-instar larvae injection, transcript levels of *Hvdyl* were measured (E). Relative transcript levels are the ratios of relative copy number in treated individuals to that of dsegfp-injected controls, which was set as 1. The values represent means (± SE). Different letters indicate significant differences at P <0.05 using t test. The three biological replicates are marked with red dots. The phenotypes of pupae (F), adults (G) and elytra (H) were observed and imaged (fourth-instar larvae injection).
